# Non invasive ventilation after extubation in paediatric patients: a preliminary study

**DOI:** 10.1186/1471-2431-10-29

**Published:** 2010-05-05

**Authors:** Juan Mayordomo-Colunga, Alberto Medina, Corsino Rey, Andrés Concha, Sergio Menéndez, Marta Los Arcos, Irene García

**Affiliations:** 1 Paediatric Intensive Care Unit. Department of Paediatrics. Hospital Universitario Central de Asturias. University of Oviedo. Oviedo. Spain

## Abstract

**Background:**

Non-invasive ventilation (NIV) may be useful after extubation in children. Our objective was to determine postextubation NIV characteristics and to identify risk factors of postextubation NIV failure.

**Methods:**

A prospective observational study was conducted in an 8-bed pediatric intensive care unit (PICU). Following PICU protocol, NIV was applied to patients who had been mechanically ventilated for over 12 hours considered at high-risk of extubation failure -elective NIV (eNIV), immediately after extubation- or those who developed respiratory failure within 48 hours after extubation -rescue NIV (rNIV)-. Patients were categorized in subgroups according to their main underlying conditions. NIV was deemed successful when reintubation was avoided. Logistic regression analysis was performed in order to identify predictors of NIV failure.

**Results:**

There were 41 episodes (rNIV in 20 episodes). Success rate was 50% in rNIV and 81% in eNIV (p = 0.037). We found significant differences in univariate analysis between success and failure groups in respiratory rate (RR) decrease at 6 hours, FiO_2 _at 1 hour and PO_2_/FiO_2 _ratio at 6 hours. Neurologic condition was found to be associated with NIV failure. Multiple logistic regression analysis identified no variable as independent NIV outcome predictor.

**Conclusions:**

Our data suggest that postextubation NIV seems to be useful in avoiding reintubation in high-risk children when applied immediately after extubation. NIV was more likely to fail when ARF has already developed (rNIV), when RR at 6 hours did not decrease and if oxygen requirements increased. Neurologic patients seem to be at higher risk of reintubation despite NIV use.

## Background

Conventional mechanical ventilation (CMV) is a core feature of intensive care. Weaning and removement of endotracheal tube are crucial processes, which often account for a considerable part of CMV total time. Unsuccessful extubation has been noted to be associated with an increase of both morbidity and mortality in adult and paediatric patients [1-4]. The documented rate of failed extubations ranges from 4.1 to 14% in paediatric intensive care units (PICUs) [1,2,5]. Therefore, strategies preventing the need for reintubation are needed.

Non invasive ventilation (NIV) has been proposed as a useful therapy to wean patients after unsuccessful weaning trials and to avoid reintubation in adults [6-10], though controversy exists at this concern [11,12]. This technique is increasingly being used in paediatric patients over the last years [13-20]. Some of these studies have included children receiving NIV because of ARF secondary to multiple causes including postextubation cases [13,14]. Other authors, however, excluded from the analysis postextubation NIV use [15-17] in accordance with NIV studies in adult patients and very low birth infants, which analyze postextubation NIV separately [6,8-10,21-23]. The reason provided to exclude these cases is that NIV characteristics are very different when applied after receiving CMV.

The objective of the present study was to determine postextubation NIV characteristics and to identify risk factors of postextubation NIV failure in children.

## Methods

A prospective observational study was conducted in our 8-bed paediatric intensive care unit (PICU) from July 2004 to December 2008 following NIV PICU protocol. To be enrolled, patients had to have undergone CMV for over 12 hours, to have adequate consciousness level (Glasgow coma score of greater than or equal to 10), to require no inotropics (or a maximum of 5 mcg/kg/minute of dopamine) and to fulfill NIV inclusion criteria, listed below. Extubation was performed following our internal PICU protocol for weaning: FiO_2 _of less than or equal to 0.4 to maintain transcutaneous oxygen saturation >94% (or baseline values in cyanotic cardiopathies), positive end-expiratory pressure (PEEP) under 8 cm H_2_O and a pressure support of under 12 cm H_2_O. An exception was made to this protocol: PEEP could be ≥ 8 cmH_2_O in children in whom an early extubation was considered beneficial due to high risk of associated-ventilator pneumonia (e.g. immunodeficiency). Sedation was progressively lowered according to our sedation protocol in order to avoid withdrawal syndrome.

Types of postextubation NIV were elective NIV (eNIV) when the patient was extubated directly to NIV, or rescue NIV (rNIV) when the child developed ARF within 48 hours after extubation.

### Inclusion criteria

• eNIV: children deemed at high-risk of extubation failure (e.g. severe kyphoescholiosis, severe neuromuscular disorders) or patients extubated from a PEEP ≥ 8 cmH_2_O.

• rNIV: ARF within 48 hours after extubation which did not respond to aggressive medical treatment and: a respiratory rate above 2 standard deviations (SD) for child's age normal range, or a partial pressure of arterial oxygen to the fraction of inspired oxygen (PaO_2_: FiO_2_) ratio under 250 and above 150 or a transcutaneous oxygen saturation <90% despite FiO_2 _of 50%, or venous PCO_2 _> 55 mmHg or arterial PCO_2 _> 50 mmHg.

### Criteria for reintubation

NIV was stopped and patients were intubated when oxygen saturation was below 90% or venous PCO_2 _above 65 mmHg despite maximal NIV setting, or if management of secretions was inadequate, or if patency of airways could not be maintained, or when any of the exclusion criteria appeared.

### Exclusion criteria

cardiorespiratory arrest, hemodynamic instability despite fluid load and vasoactive treatment, absence of cough reflex, Glasgow coma score lower than 10, vocal cords palsy, bullous neumopathy, pneumothorax and upper gastrointestinal tract active bleeding.

### Underlying condition

Patients were categorized in different subgroups according to their main underlying conditions before they were extubated. Each child could be classified in more than one subgroup. Neurologic: children with severe encephalopathy or severe neuromuscular disease; Respiratory: patients intubated due to pneumonia, asthma, bronchiolitis or with underlying bronchopulmonary dysplasia; Immunosupression; Cardiac: congenital cardiopathy; Severe scoliosis.

### Monitoring

All patients were continuously monitored by means of electrocardiography, pulse oximeter and respiratory rate. Blood gas analysis was only performed when considered necessary by the attending physician.

### NIV technique

CPAP or pressure support ventilation was delivered using a nasal mask, face mask, or helmet device. The interface was chosen according to child's age and size achieving comfortability and avoiding air leaks. When using a mask, initially it was applied manually onto the patient's face, and then it was held by a paediatric head cap. Colloid dressings were placed on the major pressure points to minimize skin injury. A heated humidifier (Fisher and Paykel Healthcare, Auckland, New Zealand) was used in all cases. Ventilators employed were BiPAP Vision (Respironics, Pittsburgh, PA) for pressure support NIV and CF 800 (Dräger, Lübeck, Germany) for CPAP.

### Ventilation strategy

In eNIV, continuous positive airway pressure (CPAP) or expiratory positive airway pressure (EPAP) was set at 1-2 cmH_2_O higher than previous PEEP during CMV. In rNIV, CPAP or EPAP initial ventilator setting was 4-5 cmH_2_O. In both types of postextubation NIV, inspiratory positive airway pressure (IPAP) was started at 6-8 cmH_2_O. IPAP was increased if the attending physician considered that inspiratory volume was low according to auscultation and thoracic motion, and if hypercarbia increased or did not decrease. CPAP or EPAP were increased if no improvement of pulse-oximetry O_2 _saturation or arterial PO_2 _was achieved. If clinical situation did not improve with CPAP and a good-fitting mask was available, the child could be switched to pressure support NIV according to the attending physician's decision.

### Data collection

Patients with multiple NIV episodes were considered individually, since each episode requiring NIV presents new variables potentially affecting outcome. For each episode, the following variables were collected: age, gender, weight, PICU stay, primary cause of intubation, underlying disease, type of postextubation NIV, paediatric risk of mortality (PRISM) score within 24 hours of admission, type of interface, NIV duration, NIV outcome, mortality and causes of death. Clinical data collected were respiratory rate (RR), heart rate (HR) and FiO_2 _before NIV was started. The same data and CPAP, EPAP and IPAP were collected at 1, 6, 12, 24 and 48 hours.

### Outcome

NIV was considered successful when reintubation was avoided.

### Statistical analysis

Mean, median, standard deviation and range were used to describe the sample. Quantitative continuous variables were compared between groups using Mann Whitney's non parametric tests if the variable had a non-normal distribution or unpaired Student's t test if the variable had a normal distribution. Qualitative variables were compared by Chi-square test (χ^2^). A logistic regression analysis was performed in order to identify possible predictors of NIV failure. Kaplan-Meier estimate-of-survival curve was used to determine the cumulative risk of avoiding reintubation. Multiple logistic regression analysis was performed before NIV start and after 1, 6 and 12 hours. Variables included in the multivariate analysis were those which had a p value under 0.2 between success and failure groups and also those variables which were considered clinically important in order to control statistical confusion. Variables included in the model were age, weight, gender, HR difference to initial HR, RR difference to initial RR, FiO_2_, neurologic condition, type of postextubation NIV. At the first hour, IPAP was also included. Before NIV was started, RR and HR were also included, instead of HR or RR difference. PO_2_/FiO_2 _ratio, PCO_2 _and pH were excluded from multivariate analysis due to the lack of data. A p value < 0.05 was considered statistically significant.

This research project was approved by the Research Ethics Committee of the Hospital Universitario Central de Asturias. Written informed consent was obtained from patients' parents or guardians.

## Results

During the study period, there were 1255 admissions in our PICU. Two hundred and thirty-eight children received CMV. Of these, 45 episodes received postextubation NIV. Three of them were excluded because they received CPAP by means of a different flow system ventilator (Infant Flow™ system). Another episode was also excluded because inclusion criteria were not fulfilled. Among those resulting 41 episodes (36 patients), 20 of them belonged to rNIV group. CPAP was only used in two cases (one failed after switching to pressure support NIV). Baseline characteristics are shown in Table 1.

**Table 1:** Baseline characteristics of the sample.

	Whole sample (N = 41)	Elective NIV (N = 21)	Rescue NIV (N = 20)	P value
**Patients' characteristics**				

Age (months)	20.9 (0.5-206.8)	15.8(0.5-195.4)	44.5 (0.8-206.8)	0.160
Males (%)	63.4	52.4	75.0	0.133
Weight (kg)	17.9 ± 16.5	13.9 ± 12.8	22.0 ± 19.1	0.124
PRISM score	11.2 ± 7.3	8.9 ± 6.4	13.6 ± 7.5	0.038
CMV duration (hours)	139.2 ± 98.4	139.9 ± 112.8	137.7 ± 81.6	0.946
HR (beats/min)	131.6 ± 30.4	129.0 ± 27.1	134.4 ± 34.0	0.580
RR (breaths/min)	32.6 ± 13.7	31.3 ± 11.7	34.1 ± 15.8	0.524
FiO_2 _(%)	36.4 ± 13.3	38.2 ± 17.1	34.5 ± 7.5	0.375
PCO_2 _(mmHg)	52.5 ± 11.2	51.4 ± 7.3	53.6 ± 13.3	0.600
PO_2_/FiO_2 _ratio	336.1 ± 146.7	291.9 ± 136.5	350.1 ± 163.1	0.614
pH	7.36 ± 0.11	7.41 ± 0.02	7.35 ± 0.12	0.525

**Underlying conditions - no**				

None	9	6	3	
Neurologic	14	6	8	
Respiratory	21	14	7	
Immunosupression	3	2	1	
Cardiac	1	1	0	
Severe scoliosis	5	1	4	

P value refers to the differences between elective and rescue NIV groups. Parameters are expressed in mean ± standard deviation except for age and CMV duration (median and range) and gender (%). Note that the addition of patients with underlying conditions exceeds the total number of patients because each patient might be included in different subgroups (see Methods section).NIV: non invasive ventilation; PRISM: paediatric risk of mortality (score); CMV: conventional mechanical ventilation; HR: heart rate; RR: respiratory rate; FiO_2_: fraction of inspired oxygen; PCO_2_: arterial partial pressure of oxygen dioxide; PO_2_: arterial partial pressure of oxygen.

**Figure 1:**
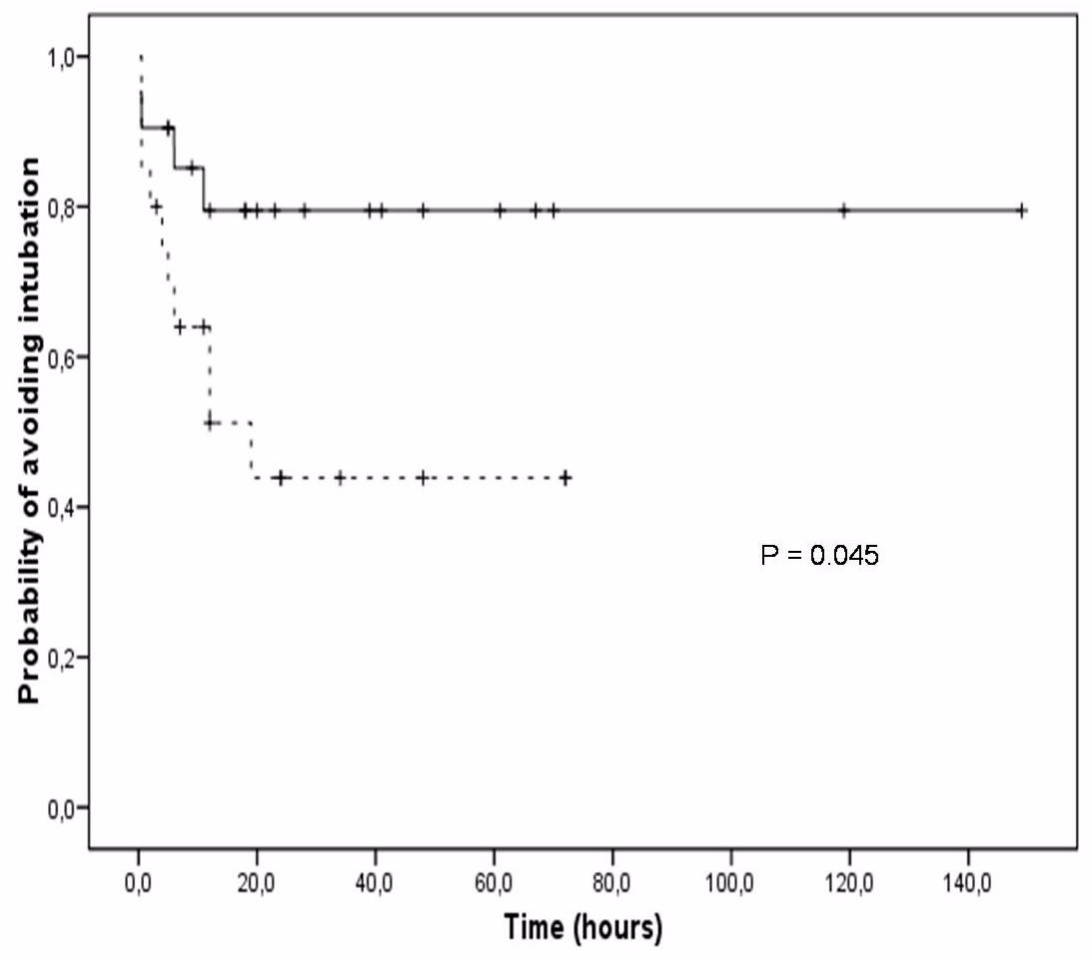
**Kaplan-Meier estimate-of-survival curve comparing the cumulative risk of avoiding reintubation regarding types of postextubation non-invasive ventilation: rNIV in discontinuous line and eNIV in continuous line**. NIV: non-invasive ventilation.

NIV was successful in 65.9% of cases. Baseline characteristics of success and failure groups are shown in Table 2. Rate of success was significantly different between rNIV and eNIV groups (50% vs 81%; p = 0.037). Figure 1 shows cumulative probability of avoiding reintubation related to rNIV and eNIV groups. In 3 episodes, extubation was performed from a PEEP over 8 cmH_2_O and eNIV was applied. None of these 3 cases failed NIV trial.

**Table 2:** Baseline characteristics of success and failure groups.

	Success group (N = 27)	Failure group (N = 14)	P value
**Patients' characteristics**			
Age (months)	30.4 (0.5 - 206.8)	11.5 (0.8 - 199.2)	0.947
Males (%)	56.0	78.6	0.096
Weight (kg)	17.4 ± 15.8	18.7 ± 18.4	0.811
PRISM score	10.5 ± 7.2	12.7 ± 7.5	0.360
HR (beats/min)	130.2 ± 30.4	134.3 ± 31.4	0.688
RR (breaths/min)	34.6 ± 14.9	28.4 ± 10.1	0.187
FiO_2 _(%)	36.0 ± 9.3	37.0 ± 19.1	0.823
PCO_2 _(mmHg)	54.6 ± 11.8	46.6 ± 3.4	0.177
PO_2_/FiO_2 _ratio	337.8 ± 168.1	284.8 ± 77.9	0.381
pH	7.35 ± 0.11	7.44 ± 0.05	0.259

**Primary cause of intubation - no (%)**			
Postsurgery	8 (29.6)	3 (21.4)	
Bronchiolitis	4 (14.8)	4 (28.6)	
Pneumonia	5 (18.5)	1 (7.1)	
Severe cranial trauma	1 (3.7)	2 (14.3)	
ARDS	2 (7.4)	0 (0)	
CPR	2 (7.4)	0 (0)	
Central apneas	0 (0)	2 (14.3)	
Upper airways respiratory infection	2 (7.4)	0 (0)	
Others	3 (11.2)	2 (14.3)	

**Underlying conditions - no (%)**			
Neurologic	6 (22.2)	8 (57.1)	0.025
Respiratory	17 (63.0)	7 (50.0)	0.424
Immunosupression	3 (11.1)	0 (0.0)	0.539
Cardiac	0 (0)	1 (7.1)	0.341
Severe scoliosis	3 (11.1)	2 (14.3)	0.768

**Type of postextubation** **NIV - no (%)**			0.037
Elective NIV	17 (81)	4 (19)	
Rescue NIV	10 (50)	10 (50)	

PRISM: paediatric risk of mortality (score); HR: heart rate; RR: respiratory rate; FiO_2_: fraction of inspired oxygen; PCO_2_: arterial partial pressure of oxygen dioxide; PO_2_: arterial partial pressure of oxygen; ARDS: acute respiratory distress syndrome; CPR: cardiopulmonary resuscitation; NIV: non invasive ventilation

Causes of NIV failure were upper airway obstruction in 5 cases, apnoeas in 3 children, hypercapnia in 2, and hemodynamic instability, hypoxemia, inability to manage secretions and massive atelectasis in 1 episode each. Median period between NIV beginning and reintubation was 4.5 hours (range 0.2 - 19 hours). Mean stay was 19.0 ± 17.7 days in success group vs. 45.3 ± 39.3 days in failure group (p = 0.001). Facial mask was used in 30 cases (13 in rNIV), nasal mask in 5 (3 in rNIV), full-face mask in 2 (1 in rNIV) and helmet in 2 (both in rNIV). Median NIV duration was 12 hours (range 0.2 - 149 hours). Two deaths occurred during the study, both children belonging to failure group. None of these deaths was related to NIV use or to NIV failure.

Table 3 shows clinical variables with statistically significant differences in univariate analysis between success and failure groups. FiO_2 _at 1 hour was found to have an area under the curve (AUC) of 0.836 to detect NIV failure. A FiO_2 _of 50% at 1 hour could predict NIV failure with a sensitivity of 76.9% and specifity of 81.5%. RR decrease at 6 hours had an AUC of 0.764 to predict NIV success. A decrease of RR at 6 hours equal to or higher than 4 bpm could predict NIV success with a sensitivity of 92.3% and specifity of 57.1%.

**Table 3:** Parameters with significant differences between success and failure groups expressed in mean ± standard deviation.

	Success group (N = 27)	Failure group (N = 14)	P value
RR decrease at 6 hours (breaths/min)	7.1 ± 9.6 (N = 27)	-4.0 ± 12.2 (N = 7)	0.015
FiO_2 _at 1 hour	36.9 ± 9.2 (N = 27)	72.7 ± 29.3 (N = 13)	< 0.001
PO_2_/FiO_2 _ratio at 6 hours	282.6 ± 74.8 (N = 8)	136.4 ± 10.8 (N = 3)	0.012

Number of episodes analyzed in each item is specified below. RR: respiratory rate; FiO_2_: fraction of inspired oxygen; PO_2_: arterial partial pressure of oxygen.

Multiple logistic regression analysis revealed that no variable included in the model was an independent predictive factor of postextubation NIV success or failure.

## Discussion

Data about postextubation NIV use in children are scarce. We conducted the present study following an observational design, as patients' management was based on our standard clinical practice criteria. To our knowledge, this is the first study to identify predictive factors of postextubation NIV outcome in a multidisciplinary PICU. Previous pediatric studies about NIV use after extubation focus on post-cardiac surgery patients [18,24].

Postextubation NIV can be used in three ways: (1) as an adjunct to weaning patients from CMV by early extubation directly to NIV, (2) as a preventive application of NIV to higher-risk patients who were extubated at the time they fulfilled standard extubation criteria, or (3) as a curative or rescue application of NIV to patients who develop ARF after having been extubated according to standard criteria. The first two indications represent children included in our study as eNIV cases. The third indication corresponds to rNIV group.

Rate of success (65.9%) was lower than rates reported in other NIV pediatric studies which do not include postextubation cases [15,17]. A very similar success rate was reported by Essouri in the subgroup of patients with respiratory failure after extubation [13] and by Pons in children after cardiac surgery [18]. This suggests that these patients must be even more carefully monitored than those with no previous CMV. Furthermore, a remarkable observation from our study was that all cases which required reintubation failed NIV trial in the first hours (Figure 1). Therefore, identification of early risk factors for NIV failure is very important in order to avoid an urgent or delayed reintubation, which seems to be associated with higher mortality in adult patients with extubation failure [25]. Conversely, if reintubation is avoided in the first 12 hours after extubation, NIV is likely to be successful.

We found that children developing ARF after extubation non-respondent to conventional medical therapy (rNIV) were more likely to fail NIV support than those extubated directly to NIV (eNIV). This goes in accordance with previous studies in adult patients. Keenan and Esteban found no outcome improvement in patients treated with NIV who developed ARF in the following 48 h after extubation when compared with standard medical therapy [8,9]. Trevisan reported the effectiveness of NIV after unsuccessful weaning trials when applied directly after extubation [6] and Nava described a lower rate of reintubation in a population considered at risk of developing postextubation respiratory failure in NIV group in comparison with standard medical therapy [7]. By contrast, our results suggest that eNIV and rNIV might be considered as two different populations regarding NIV management and effectiveness. It should be outlined that some patients in eNIV group might have avoided intubation without using NIV, but NIV's potential secondary effects are mild and NIV seems to be beneficial after extubation [18,24].

Patients with underlying neurologic conditions were found to be at higher risk for reintubation. Kurachek et al. reported the same patient feature associated with extubation failure [1]. Similar finding have been made in adult patients [26], and some reasons provided to explain this fact are pharyngeal hypotonia and diminished cough strenght and secretions clearance in these patients [1,26].

Evolution of RR in the first hours was described as an outcome predictor in several paediatric studies [13,15]. The other clinical variable identified as a risk factor in the current study was an increase in oxygen requirements. This outcome predictor has been already reported in patients with no previous CMV [15,16]. Also arterial PO_2_/FiO_2 _was significantly higher in success group in the first hour, which goes in accordance with other works in hypoxemic patients [17].

Some limitations of this study must be outlined. First, the power of the study is limited due to the sample size, which obligates us to take our results with caution. Second, it is not a randomized trial, which would be definitive to draw conclusions. Taking into account that postextubation NIV seems to be beneficial [24], performing such study could be problematic. Third, due to the fact of being an observational study, blood gas analysis were performed only if considered necessary by the attending physician. Lastly, different interfaces were used, but we consider that no NIV failure could be attributable to this. However, it must be noted that incorrect interface choosing might influence NIV outcome.

This study has several strengths. First, it is the first study focusing on paediatric postextubation NIV outcome predictors. Second, the current prospective study is based on daily clinical practice, which may allow the findings to be useful for clinical management of these children. Furthermore, despite the observational design, inclusion and reintubation criteria were well defined following our internal protocol.

## Conclusions

In conclusion, our data suggest that postextubation NIV seems to be useful in avoiding reintubation in high-risk children when applied immediately after extubation. NIV was more likely to fail when ARF has already developed (rNIV), when RR at 6 hours did not decrease and if oxygen requirements increased. Neurologic patients seem to be at higher risk of reintubation despite NIV use.

## Abbreviations

ARF: acute respiratory failure; AUC: area under the curve; CMV: conventional mechanical ventilation; CPAP: continuous positive airway pressure; EPAP: expiratory positive airway pressure; HR: heart rate; IPAP: inspiratory positive airway pressure; NIV: non invasive ventilation; PEEP: positive end-expiratory pressure; PICU: paediatric intensive care unit; PRISM: paediatric risk of mortality; RR: respiratory rate.

## Competing interests

The authors declare that they have no competing interests.

## Authors' contributions

JM-C: Data collection. Analysis and interpretation of data. Drafting of manuscript. AM: Conception and study design. Literature revision. CR: Analysis and interpretation of data. Drafting and critical revising of the manuscript. AC: Data collection and drafting of the manuscript. SM, MLA and IG: Data collection. Critical revising of the manuscript. All authors read and approved final manuscript version.

## Pre-publication history

The pre-publication history for this paper can be accessed here:

http://www.biomedcentral.com/1471-2431/10/29/prepub
